# Hepatic ductular reaction: a double-edged sword

**DOI:** 10.18632/aging.102386

**Published:** 2019-10-23

**Authors:** Tianliang Sun, Stefano Annunziato, Jan S. Tchorz

**Affiliations:** 1 Novartis Institutes for BioMedical Research, Novartis Pharma AG, Basel, Switzerland

**Keywords:** biliary epithelial cells, ductular reaction, WNT signaling, YAP signaling, cholangiopathies, liver regeneration

The liver has a remarkable regenerative capacity enabled by the plasticity of its epithelial cell compartments. Hepatocytes can re-enter the cell cycle to repopulate the hepatocyte pool or transdifferentiate into biliary epithelial cells (BECs; also termed cholangiocytes) in response to injury [1]. BECs form the bile ducts responsible for bile excretion as well as peripheral ductules and canals of Hering, where bile is collected. Upon injury activated BECs expand around the portal vein and form a transient luminal epithelium establishing an auxiliary biliary system in a process referred to as ductular reaction (DR) [2]. When hepatocyte-mediated regeneration is impaired (e.g. due to hepatocyte senescence), BECs can transdifferentiate into functional hepatocytes restoring injured liver parenchyma [3]. Despite the relevance of a DR for promoting liver regeneration, the mechanisms regulating this process were not fully understood.

Recently, we have investigated signaling pathways controlling BEC expansion using a focused CRISPR-based loss-of-function screen in mouse BEC-organoids. MTORC1, YAP signaling and WNT/β-Catenin signaling were the top hits that we identified *in vitro* and further studied in vivo during a DR, induced by DDC-mediated liver injury. While mTORC1 and YAP signaling promoted BEC expansion, and YAP induced hepatocyte-to-BEC transdifferentiation, LGR4/5-mediated WNT/β-Catenin signaling was dispensable for these processes during a DR *in vivo*. Detailed assessment of YAP and WNT/β-Catenin signaling during different steps of a DR and recovery from DDC-induced injury confirmed that YAP but not WNT/β-Catenin signaling is activated in BECs. It remains unclear why the dependency of BEC organoids on WNT/β-Catenin signaling did not translate *in vivo.* We cannot exclude that WNT/β-Catenin signaling is activated in BECs during certain conditions that our injury models did not mimic despite availability of RSPO3 and WNT ligands within the DR. However, it is also plausible that the unphysiological niche provided by Matrigel in which BEC-organoids were embedded biased their signaling in our experiments. Nevertheless, besides revealing mTORC1 and YAP as key regulators of a DR, our CRISPR screen also retrieved many common tumor suppressors in cholangiocarcinoma, which is believed to originate from malignant transformed BECs. This suggests that BEC organoids are a robust research tool to study BEC proliferation and formation of a luminal biliary epithelium *ex vivo* [4]. Back-to-back with our work, Fernando Camargo`s laboratory validated YAP as a key regulator of BEC proliferation and hepatocyte-to-BEC transdifferentiation, required for establishing a DR, and further identified a novel role for YAP in protecting BECs from apoptosis induced by cytotoxic bile during liver homeostasis [5]. Since YAP signaling promotes cholangiocarcinoma formation, fine-tuning of biliary YAP signaling seems to be essential for proper liver homeostasis [6]. Future research is needed to understand the instructive niche signals regulating YAP signaling during homeostasis, regeneration or in diverse disease settings.

Besides their important pro-regenerative functions, activated BECs within a DR have been associated with severe liver diseases such as primary sclerosing cholangitis (PSC), primary biliary cirrhosis (PBC) and other cholangiopathies. BECs can secrete pro-inflammatory factors associated with cholangiopathies and peribiliary fibrosis. Moreover, cholangiocarcinoma formation is unfortunately common in a subset of PSC patients and often only diagnosed during advanced stages [7,8]. Whether the pro-regenerative and disease-associated pathways are activated in the same BECs or in different subsets remained an important question.

**Figure 1:**
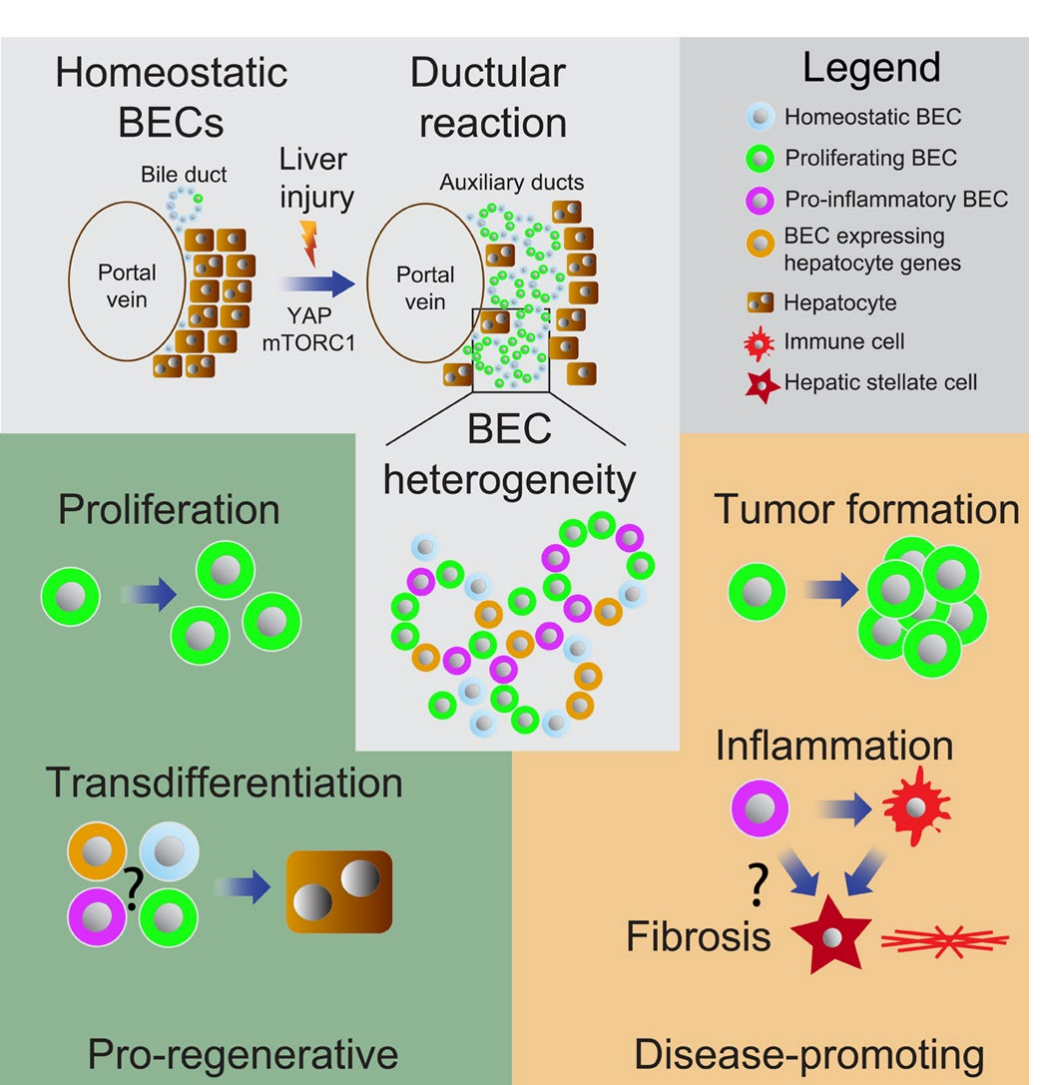
**Functional heterogeneity within biliary epithelial cell (BECs) subsets.** BECs expand around the portal vein following injury forming auxiliary ducts during a ductular reaction (DR) [2]. Single-cell sequencing revealed heterogeneity within BECs and highlighted distinct subsets with increased proliferate capacity, hepatocyte markers or expression of pro-inflammatory genes [4]. BECs can expand during DR or transdifferentiate into hepatocytes to support liver regeneration [2,3]. However, BECs can also promote inflammation and peribiliary fibrosis, and overt proliferation of malignant transformed BECs results into cholangiocarcinoma formation [7,8].

Our single cell analysis of EPCAM+ BECs within a DDC-induced DR identified three distinct subsets of BECs in mice [4]. These BEC subsets showed increased expression of genes regulating either proliferation or inflammation or had a mixed BEC-hepatocyte gene signature. Additional research is required to link these BEC subsets to the diverse functional implications these cells could have (Figure 1). It is likely that the proliferative subset we identified represents BECs currently expanding and fueling the DR, whereas BECs expressing pro-inflammatory factors might induce inflammation and subsequent activation of hepatic stellate cells (HSCs) establishing fibrosis as seen in cholangiopathies. While YAP signaling in BECs promotes the expression of secreted pro-fibrotic factors such as CTGF and CYR61 [4,6], a direct effect on HSC activation and fibrosis remains to be shown. Of note, the DDC-induced liver damage in our study was not sufficient to promote BEC-to-hepatocyte transdifferentiation. It therefore remains unclear whether the subset with a mixed BEC-hepatocyte signature represents BECs with the potential to transdifferentiate into hepatocytes or those that derived from hepatocyte-to-BEC conversion during DR. Additional single cell analyses combined with different liver injury models might reveal whether a defined subset or the majority of BECs have the ability to give rise to hepatocytes. A clear distinction of such pro-regenerative BECs from those promoting inflammation and fibrosis will likely require markers allowing their isolation and detailed characterization as the limited sequencing depth of current single cell data limits detailed signaling network analyses. Functional heterogeneity within BECs during a DR provides a new perspective to future therapeutic concepts: directed pathway modulation might allow exploiting their pro-regenerative function while blocking the signals involved in liver disease initiation and progression. Further dissecting the mechanisms establishing the differential functional identity of BECs will be key for advancing our understanding of BEC biology and for developing novel therapeutic strategies supporting liver regeneration and curing cholangiopathies.
